# Measuring adolescent girls' agency

**DOI:** 10.1002/jad.12414

**Published:** 2024-10-05

**Authors:** Bolatito O. Ogunbiyi, Jeffrey B. Bingenheimer, Sarah Baird, Amita Vyas

**Affiliations:** ^1^ Department of Prevention and Community Health, Milken Institute School of Public Health George Washington University Washington DC USA; ^2^ Department of Global Health, Milken Institute School of Public Health George Washington University Washington DC USA

**Keywords:** adolescent girls, agency, convergent validity, factor analyses, known‐groups validity

## Abstract

**Introduction:**

The last decade has experienced a surge of interventions focused on improving adolescent girls' agency. Yet measuring adolescent girls' agency continues to be a challenge, limiting the ability to track impact. This study addresses this evidence gap by constructing and validating a multidimensional measure of agency among adolescent girls in Ethiopia.

**Methods:**

This study utilized cross‐sectional data from 3033 in school adolescent girls aged 10–12 years and 15–17 years and their adult female caregivers collected as part of the Gender and Adolescent: Global Evidence study in 2017–2018 in Ethiopia. This study constructed a measure of agency among the sample and evaluated both known group and convergent validity of the scale. Twenty‐two indicators across three domains (decision‐making, voice, and mobility) were used to characterize adolescent girls' agency. The data was randomly divided into two halves for exploratory and confirmatory factor analyses separately.

**Results:**

While six factors of agency emerged from the initial exploratory analysis, two factors (decision‐making and mobility factors) defined adolescent girls' agency from the confirmatory factor analysis. Known‐groups validity of the agency scale was confirmed—the scores on the two domains (decision‐making and mobility) and the overall agency scale was higher for older girls compared to younger girls and for girls in urban households compared to those in rural households. Convergent validity of the scale was not confirmed.

**Conclusions:**

This study advances adolescent girls' agency measurement by providing a validated multidimensional measure that can be used to support future policy and research.

## 1 INTRODUCTION

Agency is defined as the capacity to make decisions about one's own life and act on them to achieve a desired outcome, free of violence, retribution, or fear (Klugman et al., 2014). Agency is not static as an individual's level of agency can change over time and in different contexts, and it is often construed as a key component of women and girls' empowerment given that it is enacted when women and girls use their resources to make decisions (Kabeer, 1999; Malhotra & Schuler, 2005; Mogford, 2011; Yount et al., 2015).

Adolescent girls make up 12 percent of the total Ethiopian population of 12 million (United Nation Population Fund, 2016). There is evidence that girls in low‐ and middle‐income countries (LMICs) are widely denied agency, or even participation in decisions such as when and whom to marry, where to go, who to spend time with, and their own health care (Banerji et al., 2008; Ross, 2011; Santhya et al., 2006, 2010). For many girls in LMICs such as Ethiopia, adolescence is a period of heightened vulnerabilities in terms of their sexual and reproductive health as it often coincides with dropping out of school, being married off very young, initiation of sexual activities, and unwanted pregnancy (Advocates for Youth, 2009; Woog & Kågesten, 2017). More than half of Ethiopian girls aged 15–19 years are sexually active and about a third have started having children during this period (Central Statistical Agency (CSA) & ICF International, 2017; Crossette & Kollodge, 2011; United Nations Population Fund, 2013). The median age at marriage is 16.5 years and 40.3% of women aged 20–24 years are married before the age 18 (CSA & ICF International, 2017). In Ethiopia, the entrenched social norms such as early marriage and prioritization of boys in education that evidently favor boys over girls diminish adolescent girls' agency and are impediments to adolescent girls improving their sexual and reproductive health (Berhane et al., 2019; French Gates, 2014; Guttmacher Institute, 2014; Tewahido et al., 2022). The existing literature shows that agency deprivation among women and girls often manifests as early marriage, early sexual initiation, unmet need for family planning, repeated unintended pregnancies, and high levels of maternal mortality and morbidity (Grépin & Klugman, 2013; Klugman et al., 2014). Agency deprivation also means that these women and girls have limited control over their sexual and reproductive rights, resulting in negative sexual and reproductive health outcomes (Darteh et al., 2019).

Among women and girls, agency has been operationalized as a multidimensional construct with three domains: decision‐making, freedom of movement or mobility, and voice or vocalization of more gender‐equitable attitudes (Kabeer, 1999; Yount et al., 2015; Zimmerman et al., 2019). Among adolescents, decision‐making is defined as the ability to make daily decisions without adult supervision or approval to young adolescents (Zimmerman et al., 2019). Freedom of movement or mobility is the ability to move freely within the environment, and voice is the ability to articulate choices and opinions (Zimmerman et al., 2019).

In more recent years, researchers have quantitatively measured agency among adolescent girls (Berhane et al., 2019; Do & Kurimoto, 2012; James‐Hawkins et al., 2018; Upadhyay & Karasek, 2012; Upadhyay et al., 2014; Yount et al., 2015; Zimmerman et al., 2019). Despite acknowledging that agency is a multidimensional construct, most of these previous studies measured agency through a single dimension such as decision‐making power and mobility (Do & Kurimoto, 2012; James‐Hawkins et al., 2018; Upadhyay & Karasek, 2012; Upadhyay et al., 2014). In addition, the existing literature shows a dearth of validated measures of agency, especially among adolescent girls (Koenig et al., 2020). Most of the evidence to date has focused on women's and girls' agency in late adolescence or adulthood, and has used measures such as autonomy, voice, self‐efficacy, or decision making as a proxy measure of agency rather than considering these proxies as a dimension of agency.

This study is informed by the Gender and Adolescence: Global Evidence (GAGE) conceptual framework which places adolescent girls within an ecological context of family or households, community, and global levels where girls at different stages of life experience different challenges, and these contextual realities in turn determine the change strategies to adopt in improving their capability outcomes (GAGE Consortium, 2019).

To our knowledge, only one study outside of our work (Zimmerman et al., 2019) has used factor analyses to explore the multi‐dimensionality of adolescent girls' agency. Zimmerman and colleagues applied exploratory factor analyses to construct and validate three subscales—voice, freedom of movement, and behavioral control and decision making—within the construct of agency among adolescents in 15 countries, including one country for validation of the subscales.

The intent of this study was to construct and validate a multidimensional measure of agency among adolescent girls in Ethiopia using exploratory and confirmatory factor analyses, contributing to the literature on the measurement of agency among adolescent girls. This paper summarizes the development of the construct of agency and it subscales, using data from Ethiopia. Additionally, we evaluated two types of construct validity of the subscales and the scale—known‐groups validity and convergent validity. Known‐groups validity is a type of construct validity that involves hypotheses pre‐specification and then testing to determine whether a tool can differentiate where differences are expected theoretically. The validity of the tool is supported if a statistical difference is reported, and if the differences are not statistically significant, either the tool or the hypothesis is flawed (Davidson, 2014; Rodrigues, et al., 2019). Evidence suggests higher level of agency among older girls compared to younger girls (Hinson et al., 2019) and among adolescents in urban compared to those in rural locations (McCarthy et al., 2022). We investigated the extent to which the subscales and the scale vary on key demographic characteristics of the girls—age and location (rural/urban), hypothesizing that the scores on each domain and the overall agency scale will be higher for older girls compared to younger girls and for girls in urban households compared to those in rural households. We also examined the expected relationships of the domains and the overall agency scale with gender attitudes and social norms of the girls, hypothesizing high positive correlation of the constructs with equitable gender attitudes and social norms.

## 2 METHODOLOGY

### 2.1 Sample description

This study utilized the publicly archived 2017–2018 baseline data from Ethiopia collected as part of the Gender and Adolescent: Global Evidence (GAGE) study (see Appendix 1 for the description of GAGE) (Baird et al., 2019; United Kingdom Data Archive, n.d.). The data from the survey instruments was downloaded from the UK data services (UK data archive, n.d.). This study covered data from a subset of the GAGE capability domains—voice, agency, and mobility; social inclusion; and gender attitudes and norms from data collected from adolescent girls in Ethiopia (Hicks et al., 2019; Hicks et al., 2020). The study sample was restricted to 3,033 never‐married, in‐school younger adolescent girls aged 10–12 and older adolescent girls aged 15–17 years (Table 1). The 10–12 years sample was drawn from both rural and urban sites, while the 15–17 years sample was only drawn from urban sites (only the older cohort was sampled in one of the sites, Batu) (Hicks et al., 2020). The data was collected through interviewers administered to the adolescents by trained field personnels.

**Table 1:** Descriptive statistics of the adolescent girls sample.

Characteristics	EFA (*n* = 1516)	CFA (*n* = 1517)	Total
Age			
10–12	1240 (81.8)	1217 (80.2)	2457 (81.0)
15–17	276 (18.2)	300 (19.8)	576 (19.0)
Total	1516	1517	3033
Residence			
Urban	479 (31.6)	489 (32.2)	968(31.9)
Rural*	1037 (68.4)	1,028 (67.8)	2065 (68.1)
Total	1516	1517	3033
Ethnicity			
Oromo	477 (34.6)	541(38.9)	1018 (36.8)
Amhara	823 (59.8)	779 (56.0)	1602 (57.9)
Others	77 (5.6)	72 (5.2)	149 (5.4)
Total	1377	1392	2769

*Note*: This table summarizes the frequency distribution (and proportion) of relevant demographic characteristics of the adolescent girls included in this study from the Gender and Adolescence: Global Evidence (GAGE) Ethiopia baseline quantitative survey. This sample is restricted to never‐married, in‐school adolescent girls with age data. The rural sample consists of only the younger cohort of adolescent girls. There are small differences in sample sizes across demographic characteristics. The sample size at the top of each column reflects the maximum sample size for that subsample.

### 2.2 Measures

Within the GAGE conceptual framework, the three domains or subscales of agency: decision‐making, freedom of movement, and voice capture different dimensions in an adolescent girl's life (GAGE Consortium, 2019). We identified 31 items related to the three domains in the core respondent survey instrument (see Appendix 2 to the full list of items and inclusion/exclusion rationale), and with further scrutiny, we reduced the items to 22 items by consolidating and dropping some items (Table 2).

**Table 2:** Stems, indicators, and response options by subdomain.

	Stem	Indicator	Response options
Decision‐making	I would like to learn how much say you think you have in the following issues in your family	How much time you spend helping around the house?	1—Not at all 2—Not much 3—A little bit 4—A great deal 99—Don't know 98—Not Applicable 97—Refused
		How much education you will get?	
		When to marry?	
		Who to marry?	
		Who you want to be friends with?	
		What to do in your free time?	
Voice	N/A	Do you feel comfortable expressing an opinion to or disagreeing with people in your age group, such as siblings and friends?	1—Yes 2—No 99—Don't know 98—Not Applicable 97—Refused
	N/A	Do you feel comfortable expressing an opinion to or disagreeing with people who are much older than you, such as parents and the elderly?	
	N/A	Do you feel that you can speak up in class when you have a comment or question?	
	Have you ever talked about [indicator] with your guardian?	Your education	1—Yes 2—No 99—Don't know 98—No guardian 97—Refused
		Work	
		When you will get married?	
		Bullying/harassment at school?	
Mobility	N/A	How many times in the past 3 months have you travelled outside of your [Kebele]/village?	1—Everyday 2—Every week at least once 3—Every 2 weeks at least once 4—Every month at least once 5—Less than once a month. 6—Never 99—Don't know 97—Refused
	In the past three months, how often have you gone to [place]?	The market	
		The homes of relatives, friends, or neighbors	
		Church/temple/mosque	
		Place in the community where you feel comfortable seeing friends (i.e., playground, sports field, open field)	
	If you were to go to [place], would you need permission from someone?	The market	1—Yes 2—No 99—Don't know 97—Refused
		The homes of relatives, friends, or neighbors	
		Church/temple/mosque	
		Place in the community where you feel comfortable seeing friends (i.e., playground, sports field, open field)	

*Note*: This table summarizes the indicators included in the exploratory factor analysis by the theorized domains. We recoded some of the response options (don't know, not applicable, no guardian, and refused) in the final analytic sample.

#### 2.2.1 Indicators for decision‐making domain

We assessed the decision‐making domain using six variables that describe the amount of say the respondent has over time spent helping around the house; the level of education to get; when to marry; who to marry; who to be friends with; and what to do in her free time. The responses to these variables include: 1—None at all; 2—Not much; 3—A little bit; and 4—A great deal of say.

#### 2.2.2 Indicators for voice domain

The 7 items we grouped under voice can be broadly classified into two categories of questions. The first category captured the respondent's perception of her voice in relation to her peers, older people and in the classroom. The response options for these items included “2—Yes” and “0—No.” The second category of items under this domain focused on the respondent's (actual) discussion of selected topics, such as education, work, when to get married and bullying in school, with her guardian. We selected the 4 items under this category from 13 questions posed separately about the respondent's discussion of the specific topics with her female and male guardian. All the items retained in the analytic sample are available among the younger and older cohorts, we generated a variable that represents the highest score between the two variables on discussing a selected topic with a female guardian and a male guardian for each topic discussed. For example, we generated a new variable on discussing education with a guardian to represent discussing education with either a female or male guardian. We generated similar variables to represent discussing about work, when to marry, and bullying/harassment in school with either a female or male guardian. The response options for the original items were “2—Yes”; “0—No”; and “1—don't know or not applicable.” The “not applicable” option applied to respondents without a female or male guardian, respectively. The value of the merged items ranged from “0” to “4,” with “0” representing “no” on the two original items, and ‘4’ representing ‘yes’ on the two original items.

#### 2.2.3 Indicators for mobility domain

The nine (9) items classified under mobility can be broadly classified into two groups. The first group of items captured the respondent's frequency of travelling out of her village; going to the market; visiting the homes of relatives, friends or neighbors; going to religious place of worship; and going to a place in the community where she feels comfortable to meet with friends. We reverse coded the response options for these items, ranging from “1—Never” to “6—Everyday,” with higher values reflecting greater frequency of visiting the selected place. The second group of items under the mobility domain focused on the need (or not) for permission to visit the different places listed above.

#### 2.2.4 Measure of gender attitudes and social norms for convergent validity

Measures of genders attitude and social norms were obtained from the core respondent survey instrument of the GAGE study. We generated a measure of gender attitudes among adolescent girls by summing the values of 23 attitudinal statements, while two social norms measures (descriptive and injunctive norms) were derived from 13 norms statements, with larger values reflecting favorable gender attitudes and social norms. The attitudinal statements measured what the respondent thinks about issues such as marriage, time allocation and education. For example, whether she thinks “a girl's marriage can wait until she has completed secondary school.” The norms statements captured descriptive norms—what the respondent thinks others do, and injunctive norms—what the respondent believes others think that she should do. Examples of descriptive and injunctive norms statements are: “most boys and girls in my community do not share household tasks equally,” and “most people in my community expect families to control their daughter's behavior more than their sons,” respectively. The gender attitudes and social norms measures were coded similarly; respondents were assigned a “2” if they agreed, a “1” if they partially agreed and a “0” if they disagreed with a favorable statement about gender equity. We reverse coded statements with a negative valence so that “2” indicated a disagreement and “0” indicated an agreement with unfavorable statements.

### 2.3 Data analysis

Data analysis was conducted using STATA Version 15.1 (Stata Corporation). We estimated relative frequencies of all items to assess their completeness and distributions. To minimize missing data in the final analytic sample, we recoded two response options—“don't know” and “not applicable”—representing a negligible number of the responses to the agency indicators. The “don't know” option for items under the decision‐making domain ranged from 0.3% to 5.8% of the responses, with two variables—respondent's say in who to marry and when to marry—having the highest proportion of don't know at over 5%. We recoded the “don't know” response option to the sample mean of the observations (Lin et al., 2016). The ‘not applicable’ response under this domain applies to girls that live independently, suggesting that girls in this category would have a lot of say on the items under the decision‐making domain. Therefore, we added the “not applicable” response option to the highest category (a great deal of say). We also recoded the “don't know” option for the first three items of voice and all the mobility items to the sample mean of the observations. Sensitivity analyses conducted on the original data set using only the sample with complete responses to all the items showed no significant differences between the recoded and the complete data sets (Appendix 3).

### 2.4 Exploratory factor analysis (EFA)

The chi‐square for the Bartlett test of sphericity for the agency indicators was significant, and the Kaiser–Meyer–Olkin test showed a score of 0.71, indicating that the correlation among the variables was adequate for factor analyses. Next, we split the data into two random halves: one half for EFA and the other half for CFA, as recommended by Worthington and Whittaker (2006), Cabrera‐Nguyen (2010), Fokkema and Greiff (2017) and Asaolu et al. (2018). We conducted exploratory and confirmatory factor analyses on two separate data sets. Given the binary or ordinal response options of the items included in the EFA and CFA, we estimated polychoric correlations in the random split samples to assess the level of bivariate association between any two items (Bandalos & Finney, 2019). These correlation matrices were the basis for the exploratory and confirmatory factor analyses.

We conducted EFA on one half of the randomly split data (*n* = 1516) to determine if the three factors or domains of agency are empirically supported. To determine the number of meaningful factors to retain in the EFA, we applied three criteria: Kaiser criterion—(eigenvalues > 1); inflection point of the scree plot; and interpretability of the factors (Costello & Osborne, 2005; Kaiser, 1960). We generated a scree plot to graphically display the additional variance explained (or eigenvalue) by adding another factor against the total number of factors and visually assess the number of factors above the natural bend or break point in the data where the curve flattens out, which will imply the ideal number of factors to be retained (Costello & Osborne, 2005). We dropped items that cross‐loaded on two of more factors (factor loading of 0.32 or higher) since there were other strong loaders on those factors (Tabachnick & Fidell, 2007). We retained items with strong factor loadings (0.40 or better) as this is an indication that a construct strongly predicts response on those indicators (Little, 2013). Next, we measured the internal reliability of the factors using polychoric ordinal alpha reliability coefficient (Gadermann et al., 2019).

### 2.5 Confirmatory factor analysis (CFA)

Subsequently, we conducted CFA on the second half of the randomly split data set to confirm the relevance of each item on the subscale and validate the domains generated from the EFA. Guided by the EFA, we excluded items that cross‐loaded on two or more factors from the CFA. To avoid an under‐identified model, we dropped any factor with fewer than three indicators as such factors are generally considered weak and unstable (Costello & Osborne, 2005).

We estimated item parameters such as factor loadings and correlations using maximum likelihood (ML) parameter estimation. We generated three goodness‐of‐fit indices; root mean square error of approximation (RMSEA) and its corresponding 90% confidence interval, comparative fit index (CFI), and the Tucker–Lewis index/non‐normed fit index (TLI/NNFI) to determine the percentage of observed measure covariation explained by the measurement model compared with the null model which accounts solely for the observed measured variances (Little, 2013). Per Hu and Bentler's recommendations, an RMSEA smaller than 0.06 and a CFI and TLI larger than 0.95 indicate relatively good model—data fit in general cited in Hu and Bentler (1999). We improved the model fit by assessing model misspecification through two criteria—the standardized residuals and the modification indices. We removed items with large standardized covariance residuals (greater than absolute 2.58) in a step‐wise manner as they affect overall model fit (Jöreskog & Sörbom, 1993). No modification index in the final CFA model was large enough to suggest model respecification (Byrne, 2012).

### 2.6 Construct validity

Furthermore, we investigated construct validity through known‐groups and convergent validity. We determined the known‐groups validity by calculating the mean scores of the subscales and the overall scale and applying two sample *t* tests to determine significant differences in the mean scores of the subscales and overall scale by age and location (rural/urban). We limited the age comparison to the two locations with older and younger cohorts. Given that the rural sample was limited to younger girls, we excluded older girls from the t‐test analysis of the constructs by location (rural/urban). Finally, we demonstrated convergent validity by applying correlation analysis to the subscales and overall scale with gender attitudes and norms girls and their female caregivers that are hypothesized to be theoretically related to the scale and subscales.

## 3 RESULTS

**Figure 1:**
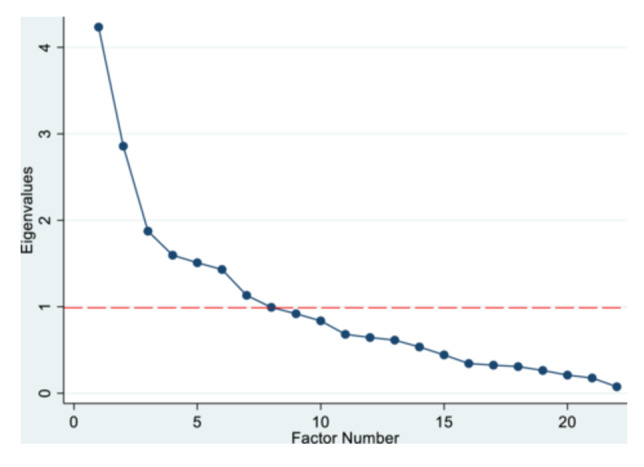
Scree plot of eigenvalues. This figure shows the scree plot from the exploratory factor analysis. The scree plot shows that the curve flattens out after the 7th factor, implying 7 as the ideal number of factors to be retained.

A scree plot showing the eigenvalues of the underlying factors derived from the EFA is shown in Figure 1. At a minimum eigenvalue of 1, the scree plot shows a natural bend on seven 7 factors. Kaiser criterion of a minimum eigenvalue of 1 also suggests 7 factors to be retained. An assessment of the interpretability of the factors based on the factor loadings and item uniqueness estimates indicate six factors to be retained. Based on the scree plot, Kaiser criterion, factor loadings and item uniqueness estimates from the EFA (Table 3), six underlying domains contribute to adolescent girls' agency in Ethiopia.

**Table 3:** Exploratory factor analysis (EFA) factor loadings for the agency scale items (*n* = 1494).

Item	Decision‐making		Voice		Mobility		Uniqueness
	Factor 1	Factor 2	Factor 3	Factor 4	Factor 5	Factor 6	
Amount of say on the time spent helping around the house	0.04	0.80^a^	−0.03	−0.13	−0.05	0.02	0.40
Amount of say on how much education you will get	0.16	0.80^a^	0.00	−0.16	0.07	0.12	0.32
Amount of say on when to marry	0.88^a^	0.09	0.05	−0.02	−0.02	0.03	0.17
Amount of say on who to marry	0.90^a^	0.03	0.07	−0.02	−0.01	0.06	0.16
Amount of say on who you want to be friends with	0.75^a^	0.12	0.08	0.08	0.01	−0.18	0.29
Amount of say on what to do in your free time^b^	0.38^a^	0.54^a^	0.10	0.03	−0.05	−0.01	0.43
Feels comfortable expressing an opinion to or disagreeing with peers	0.00	−0.19	0.06	0.88^a^	0.08	−0.01	0.27
Feels comfortable expressing an opinion to or disagreeing with older people	0.02	−0.08	0.15	0.87^a^	−0.11	0.08	0.22
Feels comfortable to speak up in class when you have a comment or question^b^	−0.33^a^	0.38^a^	0.29	0.14	−0.03	−0.02	0.68
Talked to at least a guardian about education	−0.20	0.28	0.41^a^	0.00	0.08	−0.17	0.70
Talked to at least a guardian about work	0.00	0.15	0.69^a^	0.11	0.00	0.10	0.40
Talked to at least a guardian about when you will get married	0.15	−0.02	0.80^a^	0.04	−0.06	−0.05	0.31
Talked to at least a guardian about bullying/harassment at school	0.22	−0.16	0.61^a^	0.13	0.13	−0.09	0.47
Frequency of travelling outside of [Kebele]/village	0.24	−0.12	0.06	0.05	0.03	0.57^a^	0.57
Frequency of going to the market	0.04	0.22	−0.25	0.26	0.09	0.57^a^	0.51
Frequency of going to the homes of relatives, friends, or neighbors^b^	0.04	0.19	−0.37^a^	0.35^a^	−0.04	−0.12	0.68
Frequency of going to the church/temple/mosque	−0.14	0.16	−0.05	0.16	0.04	0.65^a^	0.52
Frequency of going to comfortable place to see friends^c^	0.07	0.02	−0.10	0.26	0.09	−0.62^a^	0.52
Needs permission to go to the market	0.06	0.10	−0.16	−0.08	0.86^a^	−0.23	0.25
Needs permission to go to the homes of relatives, friends, or neighbors	−0.07	−0.05	0.13	−0.04	0.79^a^	0.21	0.23
Needs permission to go to church/temple/mosque	−0.06	−0.03	−0.05	0.10	0.90^a^	−0.10	0.21
Needs permission to go to place in the community where you feel comfortable seeing friends	0.04	−0.01	0.11	−0.05	0.79^a^	0.20	0.24
Ordinal alpha (95% CI)	0.89	0.69	0.67	0.81	0.86	0.51	
Eigenvalue	3.18	2.39	2.53	2.26	3.03	1.80	
Total % of variance explained	14.45%	10.88%	11.48%	10.29%	13.79%	8.17%	

*Note*: This table summarizes the factor loading of indicators included in the exploratory factor analysis.

Abbreviation: CI, confidence interval.

^a^ Strong factor loading.

^b^ Cross‐loaders‐ indicators that load on two or more factors at factor loading of 0.32 or higher.

^c^ Indicator with a strong negative factor loading.

The first two factors represent decision‐making related to relationships and marriage and time allocation and education, respectively. The third and fourth factors represent actual and perceived voice, respectively. The fifth and sixth factors represent perceived and actual mobility. Most of the items (18) loaded distinctly on a factor, while three (3) items loaded onto two factors with a factor loading greater than 0.34 for the second factor. One of the items (“frequency of going to comfortable place to see friends”) originally hypothesized under mobility had a strong negative factor loading on the actual mobility factor, while three other items had positive factor loading on the same factor, indicating that the item measures an opposite concept compared to the three other items.

The items loaded together as expected, however items representing decision‐making related to marriage/relationships and time allocation and education loaded on different factors, indicating two distinct types of decisions. Items representing actual and perceived voice and mobility also loaded on separate factors, indicating that actual and perceived measures of these subscales are distinct concepts in this context. The factor representing decision‐making related to relationships accounted for the largest percentage of variation in the scale (14.5%), while the factor representing actual mobility accounted for the smallest percentage of variation in the scale at 8.2%. Items that loaded at 0.32 or higher on two or more factors were dropped since there were several adequate to strong loaders (with at least 0.50) on each factor. Excluding the flagged 4 items (3 cross loaders and the item with a negative factor loading), a total of 18 items were retained in the EFA. The ordinal alpha for the subscales, calculated with items with factor loading of at least 0.4, ranged between 0.51 and 0.89.

Table 4 shows the results of the final two‐factor CFA model. Only four factors (14 items) from the EFA (Factors 1, 3, 5, and 6) were included in the initial CFA model as the other two factors (2 and 4) did not meet the factor criteria of at least three items per factor (the fit statistics of the initial four‐factor model and a one‐factor model are presented in Appendix 4). We obtained the final two‐factor CFA model by improving the model fit through two criteria—the standardized residuals and the modification indices. We removed 5 indicators (4 from the EFA factor 3 and 1 from factor 5) with large standardized covariance residuals. The results of the CFA confirmed significant and high loadings for two factors—decision‐making related to marriage/relationships and perceived mobility. In the final two‐factor CFA model, the three items on decision‐making related to marriage/relationship had significant factor loadings equal to or exceeding 0.71 on the first factor. Three mobility items had significant factor loadings of sizeable magnitude (0.61–0.94) on the second factor (mobility factor). The fit indices for the two‐factor CFA model suggest a good fit with the data.

**Table 4:** Confirmatory factor analysis (CFA) factor loadings and fit indices (*n* = 1503).

Item	Factor 1 (Decision‐making)	Factor 2 (Mobility)
Amount of say on when to marry	0.96	
Amount of say on who to marry	0.96	
Amount of say on who you want to be friends with	0.71	
Needs permission to go to the market		0.61
Needs permission to go to the homes of relatives, friends, or neighbors		0.71
Needs permission to go to place in the community where you feel comfortable seeing friends		0.94
Ordinal Alpha (95% CI)	0.98	0.92
RMSEA	0.036 (0.018, 0.054)	
CFI	0.998	
TLI	0.995	

*Note*: This table summarizes the factor loading and the fit statistics of the final CFA model.

Abbreviations: CI, confidence interval; CFI, comparative fit index; RMSEA, root mean square error of approximation; TLI, Tucker–Lewis index.

Table 5 shows the findings of the known‐groups validity. The table shows the mean value of each subscale and the overall scale by age group, and place of residence in the combined EFA and CFA subsamples. For the overall agency scale and decision‐making subscale, older girls had statistically significantly higher mean scores than younger girls. Age difference was more pronounced in the decision‐making subscale, where the average score of older girls was 7.04 and for younger girls 5.48. For the mobility subscale, the age difference was reversed with higher score noted among the younger girls compared to the older girls (5.04 compared to 4.56). Younger adolescent girls from rural locations had statistically significantly higher mean score on the decision‐making subscale than girls of the same age group from urban locations (5.78 and 5.48 respectively). On the other hand, younger adolescent girls from urban locations had significantly higher mean scores on the mobility subscale and overall agency scale than girls of the same age group from rural locations. The mobility scale demonstrated the most divergence between younger girls living in rural and urban locations, where the average score in urban locations was 5.04 and 4.23 in rural locations. Location difference was less pronounced for the overall agency, where the average score of younger girls in urban locations was 10.52 and 10.01 for younger girls in rural locations.

**Table 5:** Known‐groups validity of the decision‐making and mobility subscales and agency scale.

Item	Decision‐making	Mobility	Agency
Range (*N* = 3033)			
Mean	5.89 ± 2.78	4.40 ± 1.91	10.29 ± 3.47
Minimum	3	0	3
Maximum	12	6	18
Age (*n* = 763)			
10–12	5.48 ± 2.68	5.04 ± 1.62	10.52 ± 3.21
15–17	7.04 ± 3.22***	4.56 ± 2.02***	11.61 ± 3.63***
Location (*n* = 2457)			
Urban	5.48 ± 2.68	5.04 ± 1.62	10.52 ± 3.21
Rural	5.78 ± 2.68**	4.23 ± 1.91***	10.01 ± 3.43***

*Note*: This table summarizes the findings of the known group analysis. The table presents the mean scores of the decision‐making and mobility subscales and the overall agency scale by age and location in the combined sample (*N* = 3033). Due to the data structure, the age comparison was limited to the two locations with younger and older cohorts (*n* = 763), while the location comparison was limited to the younger cohort subsample, *n* = 2457.

** *p* < .05

*** *p* < .01.

The finding of the convergent validity of the subscales and the overall agency scales are presented in Table 6. There is a weak, however positive relationship between the scales and gender attitudes, descriptive social norms and injunctive social norms among the adolescent girls.

**Table 6:** Convergent validity of the decision‐making and mobility subscales and agency scale.

	Decision‐making	Mobility	Agency	GA	DSN	ISN
Decision‐making	1.00					
Mobility	0.07	1.00				
Agency	0.80	0.61	1.00			
GA	0.19	0.10	0.21	1.00		
DSN	0.15	0.12	0.18	0.61	1.00	
ISN	0.07	0.09	0.10	0.53	0.63	1.00
Alpha (Cronbach's)				0.35	0.60	0.47

*Note*: This table summarizes the Spearman correlation matrix of the decision‐making and mobility subscales, agency scale, gender attitudes, descriptive social norms, and injunctive social norms among adolescent girls.

Abbreviations: DSN, descriptive social norms; GA, gender attitudes; ISN, injunctive social norms.

All the correlation coefficients are significant at *p* < .001.

Table 7 shows the final measurement scale for adolescent girls' agency and scoring information for adaptation or application in future adolescent girls' empowerment programs or evaluations.

**Table 7:** Final measurement scale for adolescent girls' agency.

	Item	Response categories	Construction of the scale
**Factor 1: Decision‐making**			Sum all six items (Q1–Q6). The resulting score will range between 4 and 18, with a higher score indicating more agency.
Q1	How much say you think you have on when to marry?	1—Not at all 2—Not much 3—A little bit 4—A great deal	
Q2	How much say you think you have on who to marry?		
Q3	How much say you think you have on who you want to be friends with?		
**Factor 2: Mobility**			
Q4	If you were to go to [the market], would you need permission from someone?	1—Yes; 2—No	
Q5	If you were to go to [the homes of relatives, friends, or neighbors place], would you need permission from someone?		
Q6	If you were to go to [a place in the community where you feel comfortable seeing friends place], would you need permission from someone?		

## 4 DISCUSSION

The study confirms the contextual salience of measuring agency and raises questions on the centrality of voice in agency measurement. Six factors emerged from the initial exploratory analysis, indicating a clear divide within each originally hypothesized subscale. For example, items categorized under decision‐making loaded on decisions related to marriage and relationships and time allocation and education, suggesting that these items are influenced by two distinct constructs which we call SRH decisions and time allocation/education decisions. Similarly, our study shows a divide between items that captured respondents actual and perceived voice and mobility, indicating a clear difference in perceived and actual behavior. Similar to our findings, a recent measurement study on agency, voice, and gender attitudes among adolescents in South Asia also reported the emergence of six factors or dimensions of empowerment compared to the originally hypothesized three factors (Nagaraj, 2021).

Of the three originally hypothesized subscales, the domains describing girls' decision‐making on sexual and reproductive health issues and perceived mobility emerged as the two strongest and persistent factors from the CFA, with ordinal alphas above 0.9. Some items with high factor loading in our study were not considered central to measures of agency in earlier studies (e.g., “amount of say on the time spent helping around the house” and “talked to at least a guardian about when you will get married”), further corroborating the centrality of context in agency measurement (Richardson, 2018; Samman & Santos, 2009; Zimmerman et al., 2019). The low factor loading of some of the original items on the factors identified by the CFA could reflect the irrelevance of the items to the construct of agency. For example, the item on needing permission to go to the market loaded the lowest on the mobility subscale, suggesting a lower importance of this item to the subscale. It is possible that restricted mobility is not the latent construct influenced by needing permission to go to the market, but such permission is rather a reflection of the girls' participation in household chores.

Our findings on the voice domain departed somewhat from the work of Zimmerman and colleagues. The two initially identified domains on voice appeared be the weakest domains in the Ethiopian context, dropping completely in the CFA. Despite having a high ordinal alpha (0.81) in the EFA, the subscale on perceived voice was not included in the CFA as factors with fewer than three indicators are generally considered weak and unstable (Costello & Osborne, 2005). Our findings indicate that voice is not a relevant subscale of agency for in‐school, never‐married adolescent girls in the studied regions of Ethiopia. This finding is not surprising as some studies have differentiated between agency and voice, while maintaining the centrality of both constructs to the larger construct of empowerment (Nagaraj, 2021; Scales et al., 2010; Van eerdewijk et al., 2017). More research is needed to assess the validity of voice as an integral dimension of empowerment in the Ethiopian context.

The results of the known‐groups analysis are consistent with our conceptualization of the domains of agency. The literature suggests that the level of agency in a decision‐making process and the types of decisions made may depend on a girl's age and development stage (Hinson et al., 2019). In general, younger adolescents may be less able to exercise agency relative to older adolescents due to their nascent cognitive development stage. Further, younger adolescent girls may perceive themselves less in control of decisions around marriage and relationships compared to older girls, while decisions around education may be more relevant to younger girls (Hinson et al., 2019). Similarly, while most adolescents need parental permission to move around their communities, the evidence suggests that adolescent girls' mobility may be more restricted with age due to concerns over limited privacy, insecurity and “honor” which often lead to movement restrictions imposed on them by their parents and families (Lane et al., 2017; Plan International, 2020).

Our finding that younger girls in urban locations have higher level of agency compared to their rural counterparts aligns with a similar study among highly vulnerable girls in Zambia, which found that residence in urban areas positively predicted membership to high agency profile relative to lower agency profiles (McCarthy et al., 2022). Similarly, the results of a population level study among women of reproductive age (15–49 years) in 55 countries registered higher level of agency deprivation among women residing in rural areas compared to those in urban areas (Hanmer & Klugman, 2016). It is however worth noting that unlike the other studies, the location analysis in this study was restricted to younger girls, further highlighting that gender and age intersects with structural inequalities such as rural–urban location, in Ethiopia (Cochrane & Rao, 2019).

Our hypothesis of a high positive correlation between the dimensions of agency and equitable gender attitudes and social norms—convergent validity—was not confirmed as none of the correlation coefficients was >0.50 (Abma et al., 2016). This finding could have been due to the low reliability of the gender attitudes and injunctive social norms measures as captured by the Cronback's alpha. Despite the weak correlations, the positive relationship we found between the dimensions of agency and equitable gender attitudes and social norms is consistent with previous work on the association between agency and equitable social norms in Ethiopia (Berhane et al., 2019) and increased recognition that enhancing adolescent girls' agency can help promote equitable gender norms and attitudes (Berhane et al., 2019; Kågesten et al., 2016; Marcus & Harper, 2014).

The prevailing restrictive social norms in contexts like Ethiopia are internalized at an early age and translate to inequitable gender attitudes, which limit the opportunities for girls to exercise their agency and their ability to make the right choices at the right time (Campbell & Mannell, 2016; French Gates, 2014; Vu et al., 2016). Also, global evidence suggest that gender norms are commonly reflected in adolescent's personal gender attitudes (UNICEF, 2012). Generally, gender attitudes that endorse norms that perpetuate gender inequality are harmful to adolescent girls and invariably limit their agency (Jewkes & Morrell, 2010).

## 5 LIMITATIONS

There are five major limitations to this study. First, the use of secondary data limited the scope of indicators included in the analyses. The indicators included in the analyses could have been expanded and further refined to capture both expression and perception of agency among adolescent girls. For example, the decision‐making domain only captured perception of agency while voice and mobility items captured both expression and perception of agency. Measures of perception of agency are easily distorted by social norms and may not be comparable across individuals or countries (Klugman et al., 2014). Second, we cannot rule out social desirability bias in self‐reported agency indicators, gender attitudes, and social norms among the girls given the sensitive nature of the study and most respondents may refrain from stating the truth about their beliefs (attitudes and norms) if they believe it portrays an unfavorable image of themselves. Third, because most of the indicators included in the EFA were applicable to only in‐school and never married girls, this study could not explore agency among ever‐married and out‐of‐school adolescent girls, whereas this group of adolescent girls might actually have lower levels of agency. Fourth, the dynamic nature of agency could not be confirmed due to the cross‐sectional structure of the data. Lastly, because agency is contextual and its generalizability in different contexts is limited, the findings of this study can only be generalized to settings with similar contexts as the study population.

## 6 CONCLUSION

Our analysis confirms the multidimensionality of the concept of agency and the salience of context on how agency is measured. The factors that emerged from the exploratory and confirmatory factor analyses provide the basis to explore different dimensions of agency that are critical to positive outcomes for adolescent girls in Ethiopia and in similar LMICs with restrictive gender norms. A validated measure of adolescent girls' agency is a useful tool for global health and development researchers in designing and evaluating the impact of interventions aimed at enhancing the agency of adolescent girls. We conclude that future research should explore how indicators of expression and perception of agency adequately measure agency among adolescent girls in Ethiopia and in similar contexts. Further, more research is needed to assess the validity of voice as an integral dimension of empowerment in the Ethiopian context.

## CONFLICT OF INTEREST STATEMENT

The authors declare no conflict of interest.

## ETHICS STATEMENT

The GAGE research program was approved by the George Washington University Committee on Human Research, Institutional Review Board (071721), the ODI Research Ethics Committee (02438), the Ethiopian Development Research Institute (EDRI/DP/00689/10), the Addis Ababa University College of Health Sciences Institutional Review Board (113/17/Ext), and the Human Subjects.

## Supporting information

Supporting information.

Supporting information.

Supporting information.

Supporting information.

## Data Availability

The data that support the findings of this study are openly available in United Kingdom Data Archive at https://beta.ukdataservice.ac.uk/datacatalogue/studies/study?id=8597. Study Number 8597—Gender and Adolescence: Global Evidence: Ethiopia Baseline, 2017–2018. The data used in this publication come from Gender and Adolescence: Global Evidence (GAGE), a 9‐year longitudinal research program generating evidence on what works to transform the lives of adolescent girls in the Global South (www.gage.odi.org.uk). GAGE is funded by UK aid through a grant from the Foreign, Commonwealth & Development Office. The views expressed here are those of the author(s). They are not necessarily those of GAGE, FCDO, or other funders.
