# Supplementary material for: Measuring adolescent girls' agency
Source: J Adolesc. 2024 Oct 5;97(1):219–32. doi: 10.1002/jad.12414 (PMC11701384; doi:10.1002/jad.12414)
Supplement: Supplementary file 1 — Supporting information. [file JAD-97-219-s003.docx]

**Appendix 1: Gender and Adolescent: Global Evidence**

Gender and Adolescent: Global Evidence (GAGE) is a nine-year (2015-2024) research program, funded by UK Aid from the UK Department for International Development (DFID), that seeks to combine longitudinal data collection and a mixed-methods approach to understand the lives of adolescents in particularly marginalized regions of the Global South, and to uncover ‘what works’ to support the development of their capabilities over the course of the second decade of life, when many of these individuals will go through key transitions such as finishing their education, starting to work, getting married and starting to have children. GAGE aims to generate unique cross-country data following girls, along with their families and peers (including boys), from early adolescence through to adulthood. GAGE currently follows the lives of 18,000 adolescents, their caregivers and communities in six low- and middle-income countries in Africa (Ethiopia and Rwanda), Asia (Bangladesh and Nepal) and the Middle East (Jordan and Lebanon). GAGE measures capability outcomes such as voice and agency, and health, nutrition and SRH, contexts that shape adolescent capabilities and the change pathways such as empowering boys and girls and changing social norms (GAGE Consortium, 2019).
