# Supplementary material for: Measuring adolescent girls' agency
Source: J Adolesc. 2024 Oct 5;97(1):219–32. doi: 10.1002/jad.12414 (PMC11701384; doi:10.1002/jad.12414)
Supplement: Supplementary file 2 — Supporting information. [file JAD-97-219-s001.docx]

**Appendix 2: Selecting items for the Exploratory Factor Analysis.**

**Table A: Original stem, item and response options by sub-domain of Agency.**

| Stem | Item | Reason for Exclusion | Response Option |
| --- | --- | --- | --- |
| Domain: Participation in decision-making | | | |
| I would like to learn how much say you think you have in the following issues in your family | How much time you spend helping around the house? |  | 1 - Not at all  2 - Not much  3 - A little bit  4 - A great deal  -97 - Refused  -98 - N/A/CR lives independently  -99 - Don't know. |
|  | How much education you will get? |  |  |
|  | When to marry? |  |  |
|  | Who to marry? |  |  |
|  | Who you want to be friends with? |  |  |
|  | What to do in your free time? |  |  |
| Domain: Ease of expressing oneself | | | |
| N/A | Do you feel comfortable expressing an opinion to or disagreeing with people in your age group, such as  siblings and friends? |  | 1 - Yes  2 - No  -97 - Refused  -99 - Don't know. |
| N/A | Do you feel comfortable expressing an opinion to or  disagreeing with people who are much older than you,  such as parents and the elderly? |  |  |
| N/A | Do you feel that you can speak up in class when you have a comment or question? |  |  |
| Have you ever talked about [item] with your mother/female guardian? | Your education |  | 1 - Yes  2 - No  -97 - Refused  -98 - No female guardian  -99 - Don't know. |
|  | What you want to do for work in the future? |  |  |
|  | ~~A romantic relationship with a boy?~~ | Item dropped as it was only posed to older girls (15-17 years) |  |
|  | ~~Menstruation?~~ | Item was dropped as it was not asked about male guardians |  |
|  | When you will get married? |  |  |
|  | Bullying / harassment at school? |  |  |
|  | ~~Religion~~ | Item was dropped as it was not conceptually related to the study |  |
| Have you ever talked about [item] with your father/male guardian? | Your education |  | 1 - Yes  2 - No  -97 - Refused  -98 - No male guardian  -99 - Don't know. |
|  | What you want to do for work in the future? |  |  |
|  | ~~A romantic relationship with a boy?~~ | Item was dropped as it was only posed to older girls (15-17 years) |  |
|  | When you will get married? |  |  |
|  | Bullying / harassment at school? |  |  |
|  | ~~Religion~~ | Item was dropped as it was not conceptually related to the study |  |
| Domain: Mobility | | | |
| N/A | How many times in the past 3 months have you travelled outside of your [Kebele]/Mohalia/Village? |  | 1 - Everyday  2 - Every week at least once  3 - Every 2 weeks at least once  4 - Every month at least once  5 - Less than once a month.  6 - Never  -97 - Refused  -99 - Don't know. |
| In the past three months, how often have you gone to [place]? | The market |  |  |
|  | The homes of relatives, friends, or neighbors |  |  |
|  | Church / temple / mosque |  |  |
|  | Place in the community where you feel comfort-able seeing friends (i.e., playground, sports field, open field) |  |  |
| If you were to go to [place], would you need  permission from someone? | The market |  | 1 - Yes  2 - No  -97 - Refused  -99 - Don't know. |
|  | The homes of relatives, friends, or neighbors |  |  |
|  | Church / temple / mosque |  |  |
|  | Place in the community where you feel comfort-able seeing friends (i.e., playground, sports field, open field) |  |  |
| ~~When you are sick and want to get medical advice or treatment, is each of the following a big problem or not a big problem:~~ | ~~Getting permission to go to the doctor?~~ | Item was dropped as it was only posed to older girls (15-17 years) | ~~1 - A big problem~~  ~~2 - Not a big problem/not a problem~~  ~~-97 - Refused~~  ~~-98 - Not applicable~~  ~~-99 - Don't know~~ |

**Items in red strikethrough text were dropped*

Description: To ensure that conceptually appropriate items were selected for the exploratory factor analysis (EFA), each of the items were examined within the conceptual framework. As shown in table A above, all the original 31 items were classified under one of the three theorized domains or sub-scale: namely, decision-making, ease of self-expression and mobility.

The six items originally identified under decision-making measure perception of control of decisions related to time use, education, relationship, and marriage. These items checked out to be conceptually relevant to the sub-scale and by extension, agency, and were thus retained.

The sixteen (16) items originally grouped under ease of self-expression can be broadly grouped into four, namely: self-expression to peer; self-expression to older people; expression in the classroom; and discussion of selected topics with a guardian. The first three groups can be classified as perception of agency, while the last group can be classified as expression of agency. The first three items represent the first three groups of questions, these items were appraised to be conceptually relevant to the sub-scale and the overall construct of agency, and thus retained in the EFA. The last thirteen (items) were questions posed separately about the respondent’s discussion of specific topics with her female and male guardian. Two items on ‘discussing about a romantic relationship with a boy with a female guardian and a male guardian’ were dropped because these questions were only posed to older girls (15-17 years). Another item on discussing about menstruation with a female guardian was dropped as the question was not asked about male guardians. Lastly, two items on ‘discussing about religion with a female guardian, and a male guardian’ were dropped as these items are not conceptually related to the study. All the items retained in the analysis sample are available among the younger (10-12 years) and older (15-17 years) cohorts. Given that the conceptual focus of this research is not to underscore the differences in the discussion pattern or topics between adolescent girls and their female and male guardian, a variable that represents the highest score between the two variables on discussion with a female guardian and a male guardian was generated for each topic discussed. For example, a new variable on discussing education with at least a guardian was generated to represent discussing education with either a female or male guardian, and similar variables were generated to represent discussing about when to marry and bullying/harassment in school with either a female or male guardian. Thus, three items on expression of agency were retained under the ease of self-expression sub-scale. Ultimately, six items were retained under this domain.

The nine (10) items originally classified under mobility are measures of perception of agency and expression of agency. The items can be broadly classified into two groups: frequency of visiting important places like the market and religious place, and the need (or not) for permission to visit to these places. An item on the ease of accessing medical care when needed was dropped as it was only asked from the older girls (15-17 years). The remaining nine items were retained as they are conceptually relevant to the mobility sub-scale and agency.
