# Supplementary material for: Measuring adolescent girls' agency
Source: J Adolesc. 2024 Oct 5;97(1):219–32. doi: 10.1002/jad.12414 (PMC11701384; doi:10.1002/jad.12414)
Supplement: Supplementary file 3 — Supporting information. [file JAD-97-219-s004.docx]

**Appendix 3: Sensitivity Analysis**

**Table A: Proportion of missing data in the sample**

| Stem | Item | Percent | | |
| --- | --- | --- | --- | --- |
|  |  | Refused/ Missing | Not Applicable* | Don’t Know |
| Domain: Participation in decision-making | | | | |
| I would like to learn how much say you think you have in the following issues in your family | How much time you spend helping around the house? | 0.1 | 0.2 | 0.3 |
|  | How much education you will get? | 0.1 | 0.0 | 0.5 |
|  | When to marry? | 0.2 | 0.0 | 5.4 |
|  | Who to marry? | 0.2 | 0.0 | 5.7 |
|  | Who you want to be friends with? | 0.1 | 0.1 | 2.6 |
|  | What to do in your free time? | 0.1 | 0.1 | 1.6 |
| Domain: Ease of expressing oneself | | | | |
| N/A | Do you feel comfortable expressing an opinion to or disagreeing with people in your age group, such as siblings and friends? | 0.2 |  | 0.3 |
| N/A | Do you feel comfortable expressing an opinion to or disagreeing with people who are much older than you, such as parents and the elderly? | 0.1 |  | 0.3 |
| N/A | Do you feel that you can speak up in class when you have a comment or question? | 0.0 |  | - |
| Have you ever talked about [item] with your mother/female guardian? | Your education | 0.1 | 0.8 | 0.1 |
|  | What you want to do for work in the future? | 0.1 | - | 0.1 |
|  | When you will get married? | 0.0 | - | 0.2 |
|  | Bullying / harassment at school? | 0.1 | - | 0.1 |
| Have you ever talked about [item] with your father/male guardian? | Your education | 0.0 | 10.5 | 0.1 |
|  | What you want to do for work in the future? | 0.0 | 0.1 | 0.1 |
|  | When you will get married? | 0.1 | 0.1 | 0.1 |
|  | Bullying / harassment at school? | 0.1 | 0.1 | 0.1 |
| Domain: Mobility | | | | |
| N/A | How many times in the past 3 months have you travelled outside of your Kebele/Mohalia/Village? | 0.1 |  | 0.4 |
| In the past three months, how often have you gone to [place]? | The market | 0.1 |  | 0.2 |
|  | The homes of relatives, friends, or neighbors | 0.0 |  | 0.2 |
|  | Church / temple / mosque | 0.0 |  | 0.1 |
|  | Place in the community where you feel comfort-able seeing friends (i.e., playground, sports field, open field) | 0.1 |  | 0.2 |
| If you were to go to [place], would you need  permission from someone? | The market | 0.2 |  | 1.8 |
|  | The homes of relatives, friends, or neighbors | 0.2 |  | 0.4 |
|  | Church / temple / mosque | 0.2 |  | 1.0 |
|  | Place in the community where you feel comfort-able seeing friends (i.e., playground, sports field, open field) | 0.3 |  | 3.1 |

**Not Applicable refers to girls who live independently under the decision-making domain, and to girls without female or male guardian under the ease of expressing oneself domain.*

**Recoding the missing data**

To ensure minimum amount of missing data in the final analytic sample, two response options- ‘don’t know’ and ‘not applicable’- representing a sizable proportion of the responses were recoded. The ‘don’t know’ option for items under the decision-making domain were recoded to zero. This option ranged from 0.3% to 5.7%. of the responses, with two variables- respondent’s say in who to marry and when to marry- having the highest proportion of don’t know at over 5%. For the items on discussing selected topics with father/male guardian and mother/ female guardian, we generated a composite variable that represents the highest score between the two variables (discussion with a father/male guardian and a mother/female guardian) for each topic discussed. This also ensures that we include people who only have a single parent at home. By combining both of them, we only have 0.00% (3 observations) missing.

The ’not applicable’ response under decision-making domain applies to girls that live independently, suggesting that girls in this category would have a lot of say on the items under the decision-making domain. Therefore, we added the ’not applicable’ response option to the highest category (a great deal of say). We recoded the ‘don’t know’ option for items under the decision-making domain, the first three items of the voice domain, and all the mobility items to the sample mean of the observations (Lin, Green & Coppock, 2016). Lin and colleagues suggested that “If no more than 10% of the covariate’s values are missing, recode the missing values to the overall mean” (pg 17). Sensitivity analyses conducted on the original dataset using only the sample with complete responses to all the items showed no significant differences between the recoded and the complete datasets (Table C).

**Table C: Sensitivity Analysis with Complete Observations: EFA Factor Loadings for the Agency Scale Items (n=1326)**

| **Item** | **Decision-making** | | **Voice** | | **Mobility** | | **Uniqueness** |
| --- | --- | --- | --- | --- | --- | --- | --- |
|  | **Factor 1** | **Factor 2** | **Factor 3** | **Factor 4** | **Factor 5** | **Factor 6** |  |
| Amount of say on the time spent helping around the house | 0.05 | 0.78* | -0.01 | -0.14 | -0.01 | 0.02 | 0.43 |
| Amount of say on how much education you will get | 0.19 | 0.78* | 0.03 | -0.21 | 0.07 | 0.15 | 0.34 |
| Amount of say on when to marry | 0.90* | 0.05 | 0.04 | 0.00 | -0.02 | 0.08 | 0.15 |
| Amount of say on who to marry | 0.88* | 0.11 | 0.04 | -0.01 | -0.04 | 0.06 | 0.16 |
| Amount of say on who you want to be friends with | 0.74* | 0.14 | 0.07 | 0.10 | 0.03 | -0.18 | 0.28 |
| Amount of say on what to do in your free time^ | 0.38* | 0.57* | 0.10 | 0.04 | -0.03 | -0.05 | 0.40 |
| Feels comfortable expressing an opinion to or disagreeing with peers | 0.02 | -0.21 | 0.08 | 0.89* | 0.08 | 0.03 | 0.25 |
| Feels comfortable expressing an opinion to or disagreeing with older people | 0.04 | -0.09 | 0.14 | 0.85* | -0.11 | 0.14 | 0.24 |
| Feels comfortable to speak up in class when you have a comment or question^ | -0.30 | 0.40* | 0.32* | 0.09 | -0.08 | 0.08 | 0.68 |
| Talked to at least a guardian about education | -0.23 | 0.24 | 0.49* | 0.01 | 0.05 | -0.12 | 0.68 |
| Talked to at least a guardian about work | 0.01 | 0.16 | 0.72* | 0.09 | 0.00 | 0.05 | 0.38 |
| Talked to at least a guardian about when you will get married | 0.12 | -0.02 | 0.80* | 0.06 | -0.08 | -0.08 | 0.32 |
| Talked to at least a guardian about bullying / harassment at school | 0.21 | -0.13 | 0.61* | 0.12 | 0.15 | -0.12 | 0.46 |
| Frequency of travelling outside of [Kebele]/ Village | 0.27 | -0.13 | 0.02 | 0.06 | 0.01 | 0.58* | 0.56 |
| Frequency of going to the market | 0.03 | 0.23 | -0.22 | 0.22 | 0.04 | 0.63* | 0.50 |
| Frequency of going to the homes of relatives, friends, or neighbors^ | 0.00 | 0.29 | -0.33* | 0.32* | -0.02 | -0.07 | 0.67 |
| Frequency of going to the church/temple /mosque | -0.17 | 0.16 | -0.06 | 0.18 | 0.06 | 0.67* | 0.51 |
| Frequency of going to comfortable place to see friends^^$^ | 0.02 | 0.04 | -0.08 | 0.32* | 0.08 | -0.55* | 0.56 |
| Needs permission to go to the market | 0.02 | 0.15 | -0.17 | -0.07 | 0.90* | -0.18 | 0.18 |
| Needs permission to go to the homes of relatives, friends, or neighbors | -0.03 | -0.10 | 0.13 | -0.04 | 0.78* | 0.22 | 0.22 |
| Needs permission to go to church / temple / mosque | -0.06 | -0.01 | -0.04 | 0.11 | 0.93* | -0.10 | 0.17 |
| Needs permission to go to place in the community where you feel comfortable seeing friends | 0.03 | -0.01 | 0.13 | -0.06 | 0.78* | 0.24 | 0.21 |
| Ordinal Alpha (95% CI) | 0.90 | 0.69 | 0.67 | 0.81 | 0.88 | 0.51 |  |
| Eigenvalue | 3.24 | 2.38 | 2.70 | 2.28 | 3.15 | 1.84 |  |
| Total % of Variance Explained | 14.74% | 10.84% | 12.26% | 10.34% | 14.31% | 8.36% |  |

*Notes: This table summarizes the factor loading of indicators included in the missing data sensitivity analysis for the exploratory factor analysis. For each item, three response options- Refused, Don’t Know and Not Applicable were recoded as missing. *Strong factor loading. ^Cross-loaders- indicators that load on two or more factors at factor loading of .32 or higher. ^$^ Indicator with a strong negative factor loading.*
