# Supplementary material for: Measuring adolescent girls' agency
Source: J Adolesc. 2024 Oct 5;97(1):219–32. doi: 10.1002/jad.12414 (PMC11701384; doi:10.1002/jad.12414)
Supplement: Supplementary file 4 — Supporting information. [file JAD-97-219-s002.docx]

**Appendix 4: Fits Statistics for Alternative Confirmatory Factor Analysis Models**

|  | 4-Factor Model* | 2-Factor Model^ | 1-Factor Model |
| --- | --- | --- | --- |
| RMSEA | 0.094 (0.088, 0.099) | 0.036 (0.018, 0.054) | 0.140 (0.137, 0.144) |
| CFI | 0.894 | 0.998 | 0.657 |
| TLI | 0.864 | 0.995 | 0.606 |

**Initial model after the exploratory factor analysis.*

*^ Final model reported.*
